# Modulated Photocurrent Spectroscopy Study of the Electronic Transport Properties of Working Organic Photovoltaics: Degradation Analysis

**DOI:** 10.3390/ma13112660

**Published:** 2020-06-11

**Authors:** Emi Nakatsuka, Yo Kumoda, Kiyohito Mori, Takashi Kobayashi, Takashi Nagase, Hiroyoshi Naito

**Affiliations:** 1 Department of Physics and Electronics, Osaka Prefecture University, 1-1 Gakuen-cho, Sakai, Osaka 599-8531, Japan; e_naka3@yahoo.co.jp (E.N.); ko24be.la@gmail.com (Y.K.); kiyohito.mori.oe@pe.osakafu-u.ac.jp (K.M.); tkobaya@pe.osakafu-u.ac.jp (T.K.); nagase@pe.osakafu-u.ac.jp (T.N.); 2 Research Institute of Molecular Electronic Devices, Osaka Prefecture University, 1-1 Gakuen-cho, Sakai, Osaka 599-8531, Japan

**Keywords:** organic photovoltaics, electronic transport properties, modulated photocurrent spectroscopy, degradation

## Abstract

Electronic transport measurement using modulated photocurrent (MPC) spectroscopy is demonstrated herein in working organic photovoltaics (OPVs) before and after AM1.5G irradiation. OPVs with bulk heterojunction (BHJ) using prototypical donor and acceptor materials, poly[[4,8-bis[(2-ethylhexyl)oxy]benzo[1–2-b:4,5-b′]dithiophene-2,6-diyl][3-fluoro-2-[(2-ethylhexyl)carbonyl] = hieno [3–4-b]thiophenediyl]] (PTB7) and [6,6]-phenyl-C71-butyric acid methyl ester (PC_71_BM), were fabricated. The OPVs had inverted structures (BHJs are formed on transparent conductive oxide substrates). The photovoltaic performance of PTB7:PC_71_BM OPVs was characterized and the best power conversion efficiency was obtained at PTB7 content of 40 wt%. Electron and hole mobility were determined with MPC spectroscopy in PTB7:PC_71_BM OPVs and were well balanced at PTB7 content of 40 wt%. Degradation of the photovoltaic performance of PTB7:PC_71_BM OPVs with PTB7 content of 40 wt% caused by AM1.5G irradiation was studied. MPC spectroscopy showed that the well-balanced mobility was not affected by AM1.5G irradiation. The degradation of OPVs was not due to changes in the electronic transport properties, but mainly to the reduced short circuit current (J_sc_) and fill factor (FF). The origin of this reduction is discussed.

## 1. Introduction

Organic semiconductor thin films have unique properties such as flexibility, printability, and low cost, and organic photovoltaics (OPVs) can be formed on curved surfaces and flexible substrates for the power sources of mobile devices and applied to light sensing for biological sensors [1]. High power conversion efficiency (PCE) has been achieved using bulk heterojunction (BHJ), a mixture of donor and acceptor organic semiconducting materials [1]. Recently, the PCE of OPVs has been remarkably improved (∼14%) [2], mainly because of the development of non-fullerene electron acceptors [3]. Electronic transport properties of BHJ have a strong impact on the photovoltaic performance; PCE exhibits the maximum value when electron mobility is equal to hole mobility (electron and hole mobility are balanced) in BHJ [4]. PCE is further increased by this increased balanced mobility. Measuring electron and hole mobility is thus fundamentally important, and generally steady-state trap-free space-charge-limited current (SCLC) expression has been applied to determine electron and hole mobility in electron-only and hole-only devices (EODs, HODs) of BHJ [5]. However, the standard SCLC technique cannot be applied to mobility measurement in working OPVs, hence in degraded working OPVs, because the electronic transport properties caused by photo-induced degradation in working OPVs are not necessarily the same as those in EODs and HODs.

In a conventional OPV cell configuration, the anode layer typically consists of a thin layer of indium tin oxide (ITO) coated with a p-type interface layer of poly(3,4-ethy lenedioxythiophene):poly(styrene sulfonate) (PEDOT:PSS). ITO is frequently used because it is conductive, transparent, and has a high work function. As a hole transporting layer, PEDOT:PSS forms an ohmic contact with BHJ. The cathode materials in a conventional OPV are typically low-work function metals such as calcium, aluminum, and magnesium. Since low-work-function materials are easily oxidized when exposed to air, OPVs with cathodes must be encapsulated to avoid air exposure.

The inverted OPV configuration reverses the conventional OPV layer sequence with respect to the ITO substrates to avoid the use of easily oxidized metal cathodes, improve device stability, and improve overall device performance [6,7]. A layer of a low-work-function material is deposited directly onto the ITO electrode surface to form the electron transport layer (ETL), thus converting the ITO to a cathode. Typical ETL materials used in inverted OPVs include cesium carbonate (Cs_2_CO_3_), and n-type metal oxides such as titanium oxide (TiO_x_) and zinc oxide (ZnO). The anode interlayer, or hole transporting layer, is most often fabricated from PEDOT:PSS or one of many high-work-function transition metal oxides, including MoO_3_, WO_3_, and V_2_O_5_ [8]. Air-stable anodes such as Ag and Au can be used in inverted OPVs. Many materials used in inverted OPVs can be processed in solution, which notably lowers the fabrication cost because a vacuum system is not needed, and a roll-to-roll printing process is available. In fact, several types of solution process for fabricating OPVs have already been demonstrated, including spray coating [9], gravure printing [10], flexographic printing [11], screen printing [12] and electrospray deposition [13].

The device stability of inverted OPVs is improved compared to conventional OPVs, as mentioned above. However, the degradation of photovoltaic performance of inverted OPVs is still a challenging issue. Degradation mechanisms in OPV are generally complicated and include a variety of processes: photo-bleaching of the photoactive layer and trap generation [14,15], degradation of the hole conducting PEDOT:PSS layer [16], ion migration from the electrodes, and morphological changes of the device [14]. These processes are induced simultaneously by exposing OPVs to sunlight and therefore are almost inseparable. This complicates the task of revealing the processes responsible for specific degradation phenomena.

In this paper, we study the degradation of electronic transport properties of inverted OPVs before and after simulated sunlight (AM1.5G) exposure by means of a modulated photocurrent spectroscopy (MPC) technique [17]. We have shown that MPC techniques can be applied to the simultaneous determination of electron and hole mobility in working OPVs with prototypical BHJ. It was therefore expected that the degradation of electronic transport properties in working OPVs could be studied separately. Poly(3-hexylthiophene-2,5-diyl) (P3HT):phenyl C_61_ butyric acid methyl ester (PCBM) and pcoly[[4,8-bis[(2-ethylhexyl)oxy]benzo[1–2-b:4,5-bB]dithiophene-2,6-diyl][3-fluoro-2-[(2-ethylhexyl) carbonyl]thieno[3–4-b]thiophenediyl]] (PTB7):phenyl C_71_ butyric acid methyl ester (PC_71_BM) [1] are known as prototypical BHJs. Since the device lifetime of inverted OPVs with PTB7:PC_71_BM BHJ is shorter than that of inverted OPVs with P3HT:PCBM BHJ and the degradation process of PTB7:PC_71_BM OPVs can be easily observed, we studied the degradation process of inverted OPVs with PTB7:PC_71_BM BHJ.

## 2. Modulated Photocurrent Spectroscopy

The expression of MPC can be obtained by solving the current continuity Equation:

(1) $$\mu_{0}F\frac{\partial n(x,t)}{\partial x}+\frac{\partial n(x,t)}{\partial t}=G(x,t)$$

where $\mu_{0}$ is charge carrier mobility, F is the electric field in the BHJ of an OPV, n(x,t) is charge carrier density at position x and time t, and G(x,t) is photogenerated charge carrier density. The equation is solved under small signal condition (*F* is uniform), with the contact at x=0 blocking and the photocarriers generated uniformly throughout the OPV. We assume $G\left(x,t\right)=G_{1}\exp\left(-i\omega t\right)$ and $n\left(x,t\right)=n_{1}\left(x\right)\exp\left(-i\omega t\right)$, and substitute G(x,t) and n(x,t) into the continuity equation. We then obtain MPC as

(2) $$J(\omega )=\frac{qLG_{1}}{\omega^{2}t_{t}^{2}}\left[1-i\omega t_{t}-\exp(-i\omega t_{t})\right]$$

from

$$J(\omega )=\frac{qF}{L}\int_{0}^{L}n_{1}(x)dx$$

where *q* is the electronic charge and *L* is the thickness of the BHJ of the OPV. In Equation (2), $t_{t}$ is the charge carrier transit time, expressed as

(3) $$t_{t}=\frac{L}{\mu_{0}F}=\frac{L^{2}}{\mu_{0}\left|V-V_{bi}\right|}$$

where *V* is the voltage applied to the OPV and *V_bi_* is the built-in voltage of the OPV.

As shown numerically and experimentally, we generally observe two peaks, one due to electron transit and the other to hole transit, in the imaginary part of J(ω), Im[J(ω)]; and the peak frequency, $f_{\max}$, is related to $t_{t}$ via $t_{t}={\left(2f_{\max}^{}\right)}^{-1}$. The value of μ is thus obtained from

(4) $$\mu_{0}=\frac{2L^{2}f_{\max}}{\left|V-V_{bi}\right|}$$

We stress that charge carrier mobility can be determined in working OPVs and from the peak frequencies in Im[J(ω)] spectra in either nondispersive or dispersive transport [18]. In other words, the peaks in the spectra are observed even in the presence of localized states.

## 3. Experiment

### 3.1. Solar Cell Fabrication and Characterization

**Figure 1:**
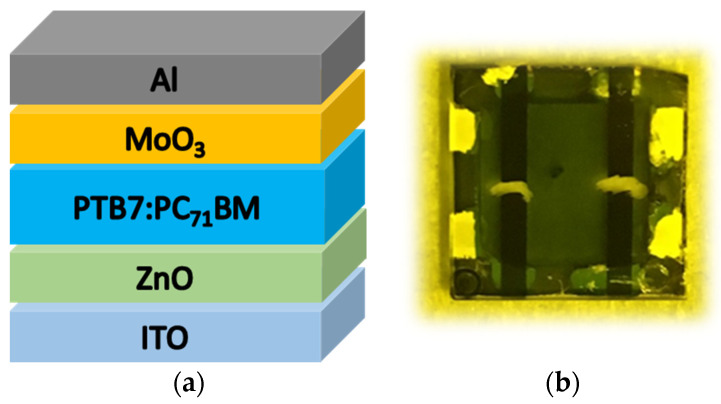
(**a**) Schematic illustration of device structure of inverted OPV and (**b**) photograph of inverted OPV cell.

Inverted OPVs with an effective area of 4 mm^2^ were fabricated on ITO-coated glass substrates (Geomatec) with a 2 mm stripe pattern. The device structure was ITO/ZnO (50 nm)/PTB7:PC_71_BM (100 nm)/MoO_3_ (10 nm)/Al (50 nm), as shown in Figure 1 (a photograph of the inverted OPV cell is also shown), where PTB7 and PC_71_BM were obtained from 1-Material (OS0007 and OS0633, respectively). The numbers in parentheses represent the thickness of the layers, and the thickness of ZnO and PB7:PC_71_BM was measured by means of a stylus profiler (Alpha-Step, Tencor: Milpitas, CA, USA) and that of MoO_3_ and Al by means of a quartz crystal oscillation type deposition controller (CRTM-9200, Ulvac: Kanagawa, Japan). A patterned ITO glass that was used as a cathode was cleaned using acetone, 2-propanol, and deionized water and then by an ultraviolet (UV)-ozone method. Subsequently, a layer of thin ZnO nanoparticles (Sigma-Aldrich, 793361-25ML: St. Louis, MO, USA) was spin-coated onto the ITO glass surface at 1000 rpm for 60 s. The substrate was then annealed in the ambient atmosphere for 10 min at 200 °C. The BHJ layer was spin-coated onto the ZnO layer from chlorobenzene solution containing PTB7 and PC_71_BM at a spin rate of 800 rpm. The weight ratio of PTB7 to mixed PTB7 and PC_71_BM solute was changed from 20 to 60 wt%. The mixing ratio of PTB7 was varied while the concentration of solution was kept constant, so that the resultant BHJ layer thickness stayed almost the same. Then, 2 wt% (PTB7 and PC_71_BM) solute with different ratios of PTB7 was dissolved in chlorobenzene, and 3 vol% 1,8-diiodooctane was dissolved in the chlorobenzene solutions. After deposition of the BHJ layer, the BHJs were dried for 60 min at 25 °C and then annealed for 10 min at 150 °C. MoO_3_ and Al layers were then thermally evaporated successively onto the BHJ layer in a vacuum chamber at a base pressure of 10^−3^ Pa. All fabrication processes were done in a glove box filled with nitrogen gas (dew point of −80 °C), and the OPVs were taken from the glove box after the encapsulation. The current density–voltage characteristics were recorded with a source meter (Wacom, IV02110-07AD1NK: Saitama, Japan) under 100 mWcm^−2^ AM1.5G irradiation from a solar simulator (Wacom, WXS-155S-10: Saitama, Japan).

### 3.2. MPC Measurements

**Figure 2:**
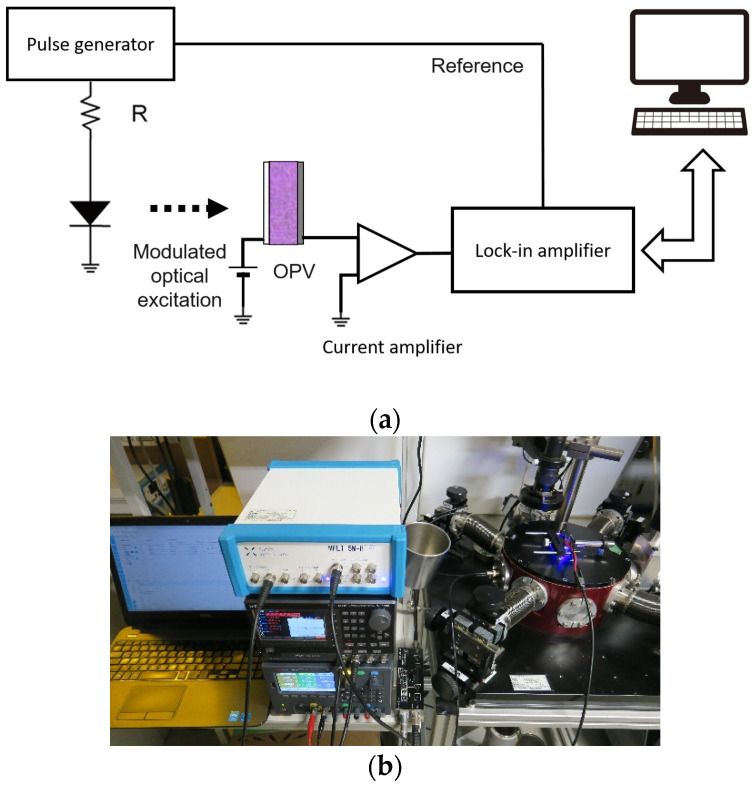
(**a**) Block diagram and (**b**) photograph of experimental setup of MPC spectroscopy.

Modulated light with 470 nm emission from a light-emitting diode was irradiated through the ITO substrates of OPVs under different biasing conditions. The resulting MPC was detected using a current amplifier (FEMTO, DHPCA-100: Berlin, Germany) and a lock-in amplifier (Zurich Instruments, MFLI 5M-H: Zurich, Switzerland). OPVs were held in a probe station (Thermal Block, SB-LN2PS: Saitama, Japan), and MPC measurements were carried out at 25 °C. A block diagram and photograph of the experimental setup are shown in Figure 2.

## 4. Results and Discussion

### 4.1. OPV Performance

**Figure 3:**
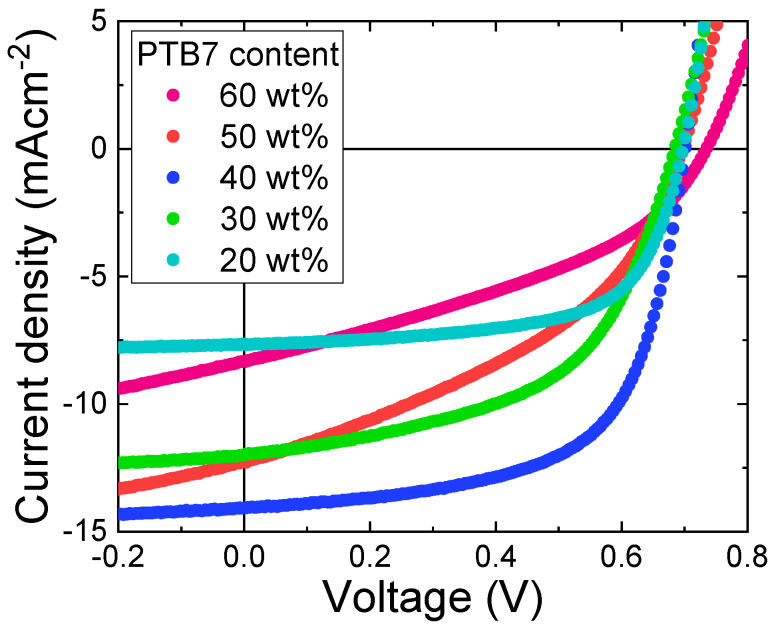
Current density–voltage (J–V) characteristics of PTB7:PC_71_BM inverted OPVs with different PTB7 content under 100 mWcm^−2^ AM1.5G irradiation at room temperature.

**Figure 4:**
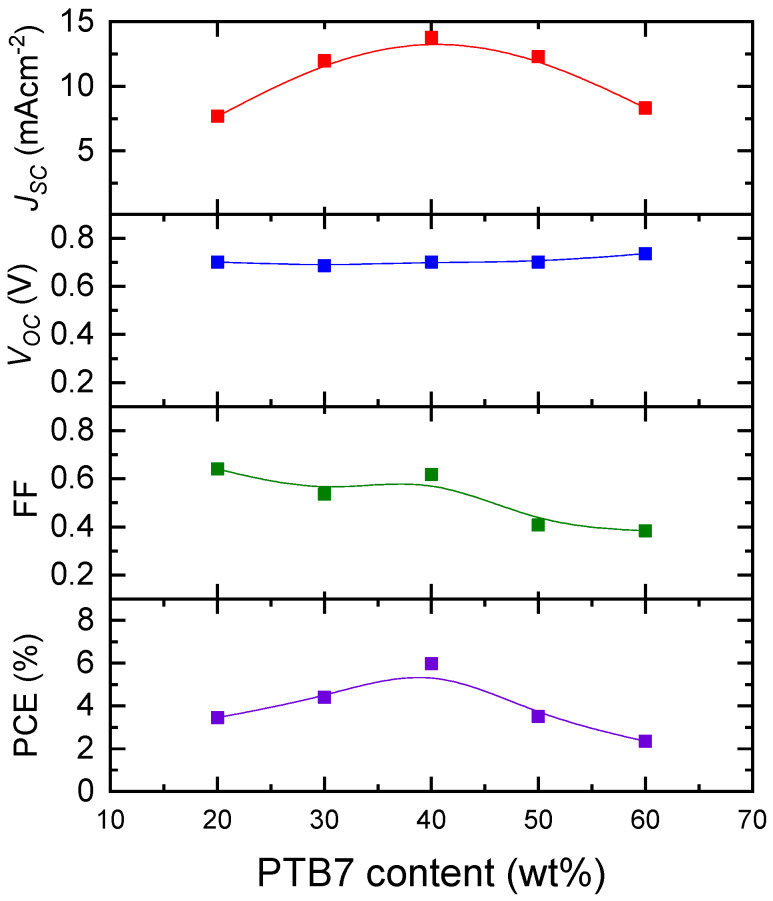
Dependence of photovoltaic performance (V_oc_, J_sc_, FF, and PCE) of PTB7:PC_71_BM inverted OPVs on PTB7 content.

Photovoltaic properties of OPVs with a structure of ITO/ZnO/PTB7:PC_71_BM/MoO_3_/Al were studied at different PTB7:PC_71_BM weight ratios. PTB7 acts as the electron donor while PC_71_BM acts as the electron acceptor. The optimized thickness of the BHJ of OPVs with different PTB7:PC_71_BM weight ratios was about 100 nm. Figure 3 shows current density–voltage (J–V) characteristics of the OPVs under the illumination of AM1.5G, 100 mWcm^−2^. The solar-cell performances obtained from Figure 3 are shown in Figure 4.

The open circuit voltage (V_oc_) is almost independent of PTB7 content. In contrast, distinct peaks were observed for the short-circuit current density (J_sc_), the fill factor (FF), and PCE at PTB7 content of 40 wt%, which is consistent with what was reported in the literature in PTB7:PC_71_BM OPVs [19]. PCE, FF, and J_sc_ of 40 wt% PTB7 OPVs are 6.2%, 63%, and 13.5 mAcm^−2^, respectively.

### 4.2. MPC Spectra of Working OPV

**Figure 5:**
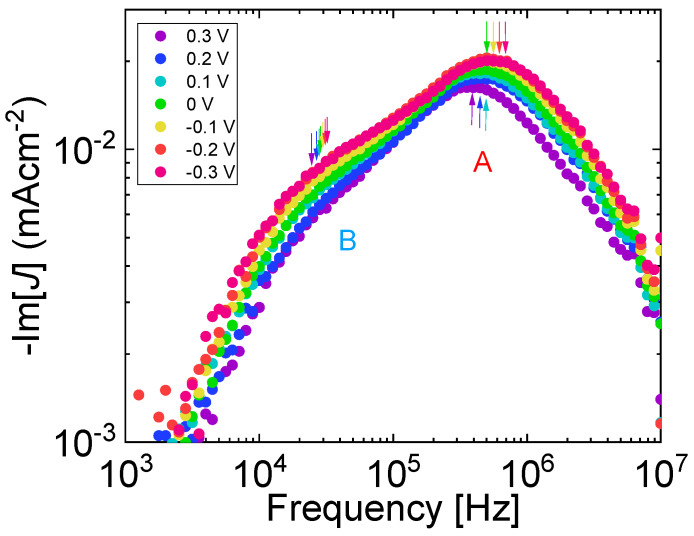
Imaginary part of MPC spectra of 60 wt% PTB7:PC_71_BM inverted OPVs at different applied voltages. Minus signs mean reverse bias voltage. Arrows highlight frequencies from which charge carrier transit times were calculated.

**Figure 6:**
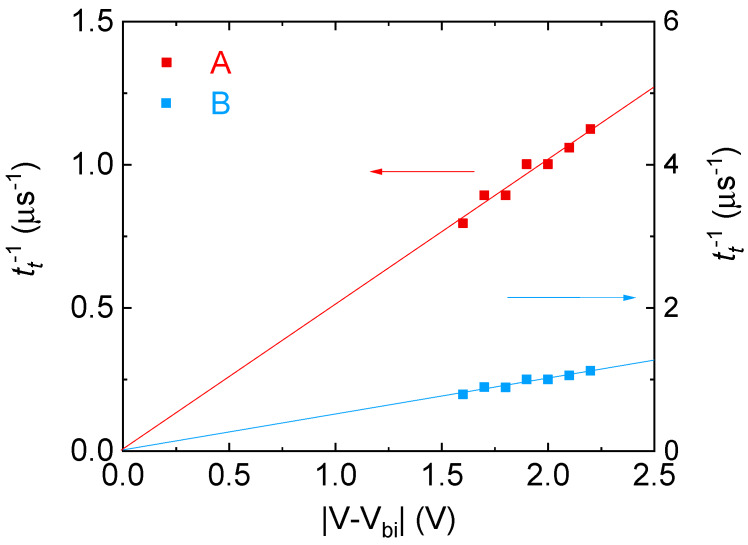
Effective applied voltage dependency of inverse transit times calculated from frequency regions of Figure 5.

Figure 5 shows MPC spectra of OPV with PTB7 content of 60 wt%. Two structures, a shoulder at (2–4) × 10^4^ Hz and a peak at (3–5) × 10^5^ Hz, are clearly seen and the structures are shifted to higher frequency regions with increasing effective applied voltage. The transit times were calculated from the frequencies of the two structures, and the inverse transit times against the effective applied voltage are shown in Figure 6. The inverse transit times of the two structures are proportional to the effective applied voltages, indicating that the structures are due to the charge carrier transit. The slopes of the straight lines in Figure 6 give charge carrier mobility of 5.1 × 10^−5^ cm^2^V^−1^s^−1^ from the peak and 1.4 × 10^−6^ cm^2^V^−1^s^−1^ from the shoulder. Figure 6 demonstrates that the simultaneous determination of electron and hole mobility can be made in working OPVs.

**Figure 7:**
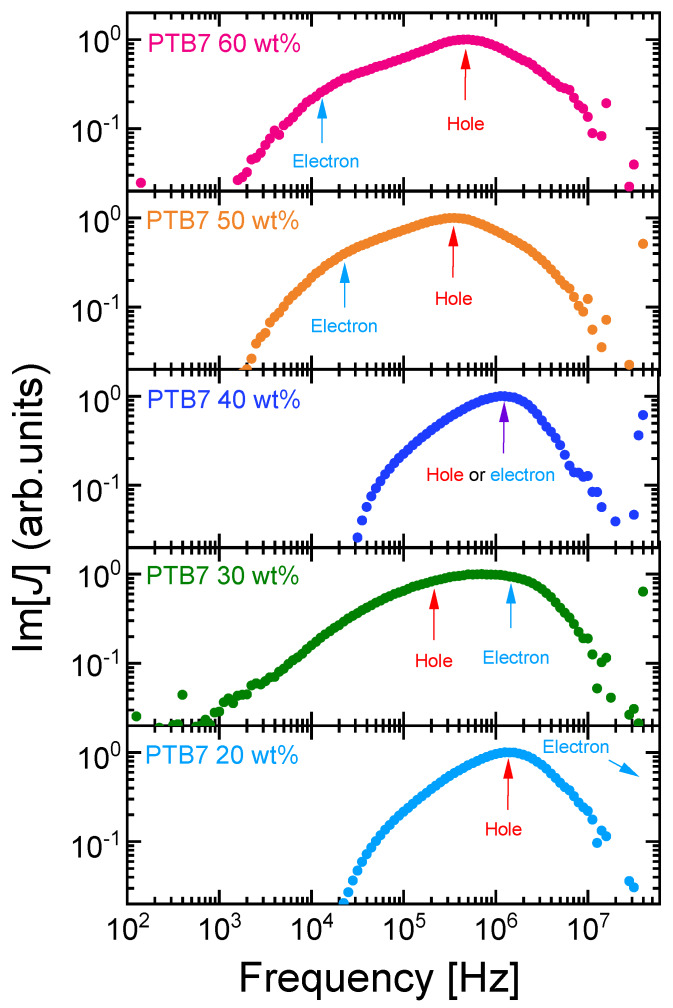
Imaginary part of MPC spectra of PTB7:PC_71_BM inverted OPVs with different PTB7 content under short-circuit conditions. Arrows highlight frequencies at structures due to electron and hole transit. Structure due to electron transit in 20 wt% PTB7:PC_71_BM OPV is not observed and may be located at a higher frequency (>10 MHz).

**Figure 8:**
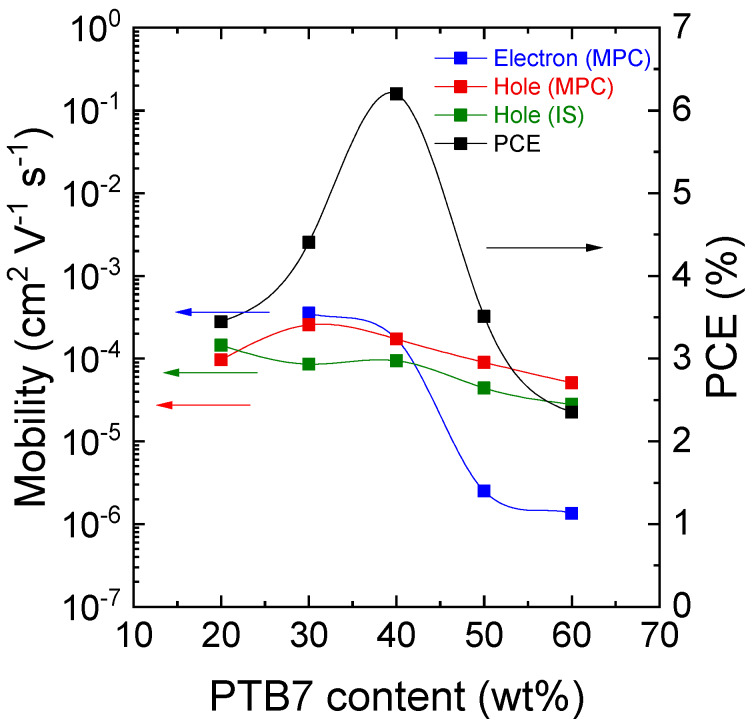
PTB7 content dependency of electron and hole mobility and PCE. Hole mobility determined by impedance spectroscopy (IS) is also shown.

Electron and hole mobility in OPVs can be assigned by examining the PTB7 content dependence of MPC spectra. Figure 7 shows MPC spectra at different PTB7 content from 20 to 60 wt%. Two structures are clearly seen in OPVs with PTB7 content of 30, 50, and 60 wt%, while a single peak is observed in OPVs with PTB7 content of 20 and 40 wt%. Although two structures are less clearly seen in the MPC spectrum of OPVs with PTB7 content of 30 wt%, they can be identified from applied voltage dependence of the frequencies at the structures. We calculated the mobility from the frequencies at the structures in Figure 7 in PTB7:PC_71_BM OPVs. Electron and hole mobility in PTB7:PC_71_BM OPVs with different PTB7 content are shown in Figure 8. The mobility exhibiting strong anticorrelation with PTB7 content (i.e., strong correlation with PC_71_BM content) was assigned as electron mobility. Such PTB7 content dependency of electron and hole mobility as assigned is consistent with that reported in the literature [19], in which electron and hole mobility were determined from SCLC measurements of EODs and HODs of PTB7:PC_71_BM BHJ with different PTB7 content. The PTB7 content dependence of hole mobility is also consistent with that determined by impedance spectroscopy measurements [20,21,22] in PTB7:PC_71_BM OPVs, as shown in Figure 8. Contrary to PTB7 content dependence of electron mobility, the addition of PC_71_BM has almost no impact on hole mobility. The content dependence of hole mobility in Figure 8 is not similar to that observed in another prototypical BHJ OPV, P3HT: PCBM, in the sense that the addition of PCBM has a strong impact on hole mobility in P3HT:PCBM OPVs [18].

As mentioned above, a single peak in the MPC spectra is observed in OPVs with PTB7 contents of 20 and 40 wt%, as shown in Figure 7. This is because the electron mobility is equal to the hole mobility in OPVs with PTB7 content of 40 wt%, which is obvious from the SCLC measurements of EODs and HODs of PTB7:PC_71_BM BHJ [19]. In 20 wt% PTB7:PC_71_BM OPVs, the frequency peak due to hole transit is observed, while the frequency peak due to electron transit may be located above 10 MHz, which cannot be resolved with the present measurement system.

The PTB7 content dependencies of electron and hole mobility in Figure 8 are fundamentally different. PTB7 content dependency has been discussed in the literature [19]. Hole conduction paths are along or between PTB7 chains, which act like conduction networks. PTB7 chains are generally better connected, and therefore hole mobility is insensitive to PTB7 content in PTB7:PC_71_BM BHJ. On the other hand, electron conduction takes place between PC_71_BM domains, which can be modeled as small nanoparticles. A drastic increase in electron mobility against PC_71_BM content is likely to be due to electron conduction in a cluster formed by the small nanoparticles from below to above the percolation threshold [23]. An illustration of electron and hole conduction paths in PTB7:PC_71_BM BHJ with different PTB7 content is depicted in the literature of [19].

PCE in Figure 8 exhibits the highest value at PTB7 content of 40 wt%, where electron and hole mobility are balanced, consistent with the results of device simulation [4]. Electron and hole mobility are well-balanced in the PTB7 content range of 30–40 wt%, while J_sc_ exhibits the maximum value at a PTB7 content of 40 wt%. In addition to the well-balanced mobility, the photocarrier generation is attributable to the maximum PCE of PTB7:PC_71_BM OPVs.

### 4.3. Degradation of OPV Performance under AM1.5G Irradiation

**Figure 9:**
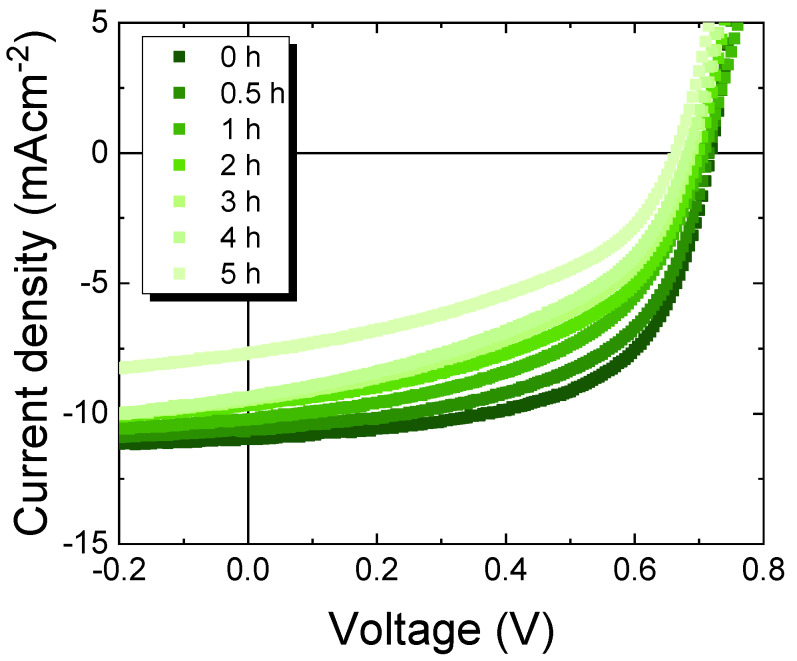
J–V characteristics of 40 wt% PTB7:PC_71_BM inverted OPV under 100 mWcm^−2^ AM1.5G irradiation at different stages of photo-induced degradation.

**Figure 10:**
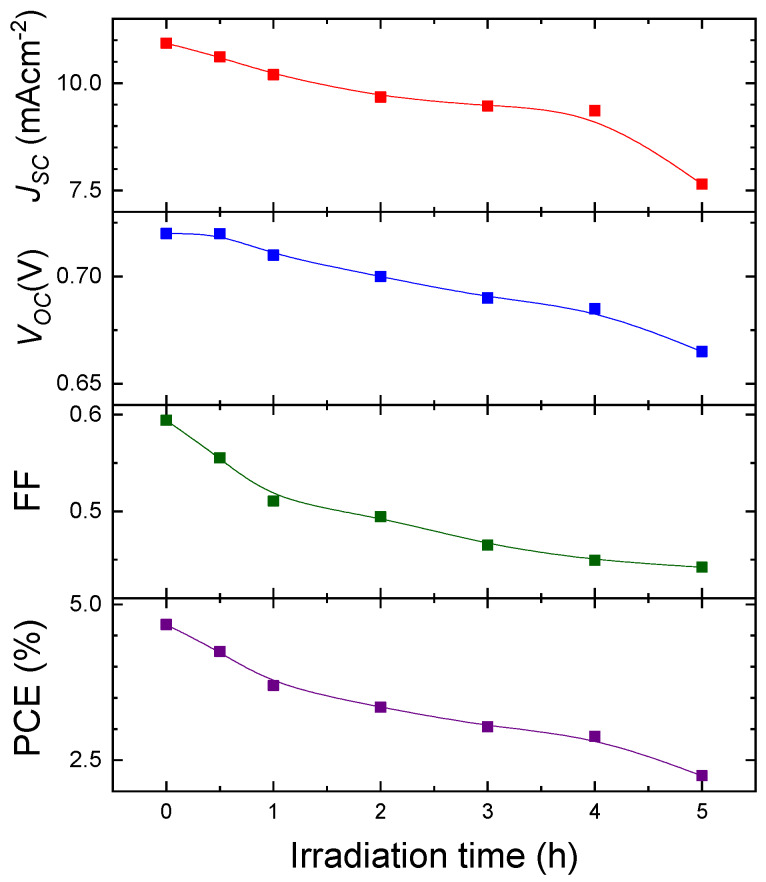
Photo-induced degradation of photovoltaic performance (J_sc_, V_oc_, FF, and PCE) of 40 wt% PTB7:PC_71_BM inverted OPV under 100 mWcm^−2^ AM1.5G irradiation as a function of irradiation time.

Degradation of solar-cell performance was observed in PTB7:PC_71_BM OPVs with PTB7 content of 40 wt%, at which the best PCE was observed. The OPVs were continuously exposed at room temperature, under open circuit or short circuit conditions, to 100 mWcm^−2^ AM1.5G light for 0–5 h, while the J–V characteristics were measured at constant time intervals throughout the degradation experiment. Figure 9 shows the J–V characteristics of the OPVs at different AM1.5G light soaked times under short circuit conditions. The photoinduced degradation behavior is shown in Figure 10, which shows the time evolution of the main solar cell parameters (V_oc_, J_sc_, FF, and PCE). All parameters in Figure 10 degrade. The degradation of PCE is due mainly to the reduction in J_sc_ and FF to 70% and 72% of their initial values, respectively. Photoinduced degradation similar to that in Figure 9; Figure 10 was observed in the OPVs degraded under open circuit conditions. The photoinduced degradation behavior shown in Figure 9 and Figure 10 was also found in five PTB7:PC_71_BM OPVs with PTB7 content of 40 wt%. Such photoinduced degradation of PTB7:PC_71_BM OPVs under light irradiation was reported in the literature [24]; the temporal changes in the main solar cell parameters induced by 5 h AM1.5G irradiation shown in Figure 10 are almost the same as those reported in the literature [24], in which the device structure of PTB7:PC_71_BM inverted OPVs is the same as in the present study.

### 4.4. Electronic Transport Properties in Degraded OPVs

**Figure 11:**
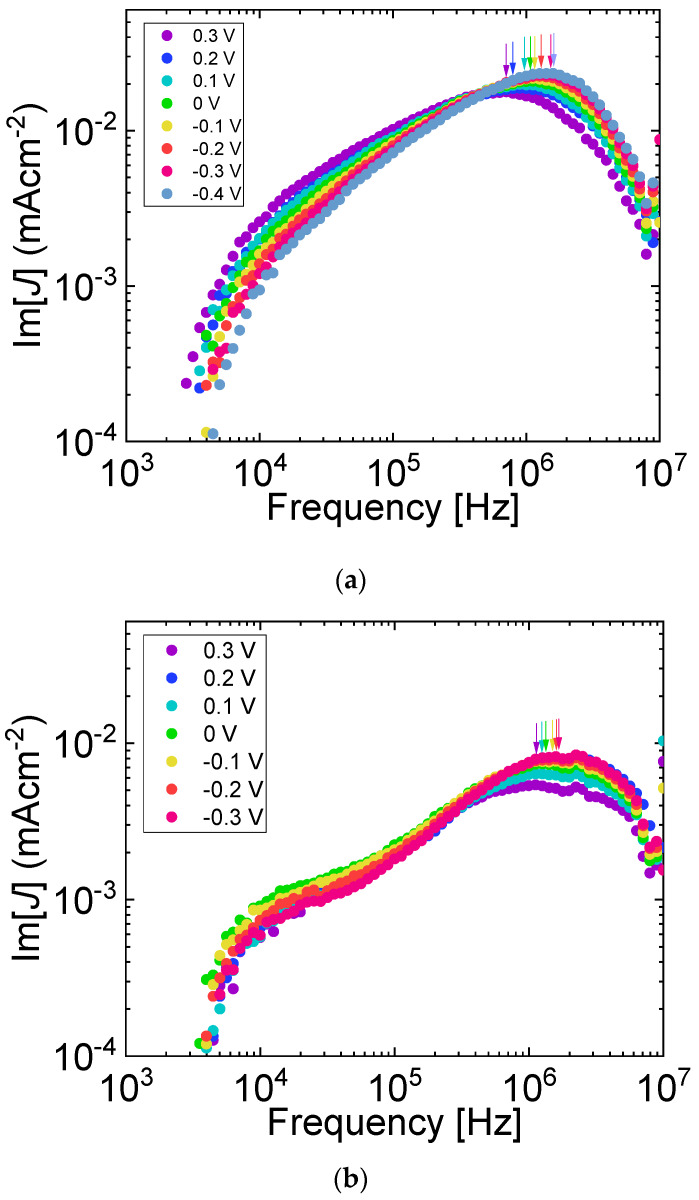
Imaginary part of MPC spectra of 40 wt% PTB7:PC_71_BM inverted OPV at different applied voltages (**a**) before and (**b**) after 5 h of photoinduced degradation. Arrows highlight frequencies from which charge carrier transit times were calculated.

**Figure 12:**
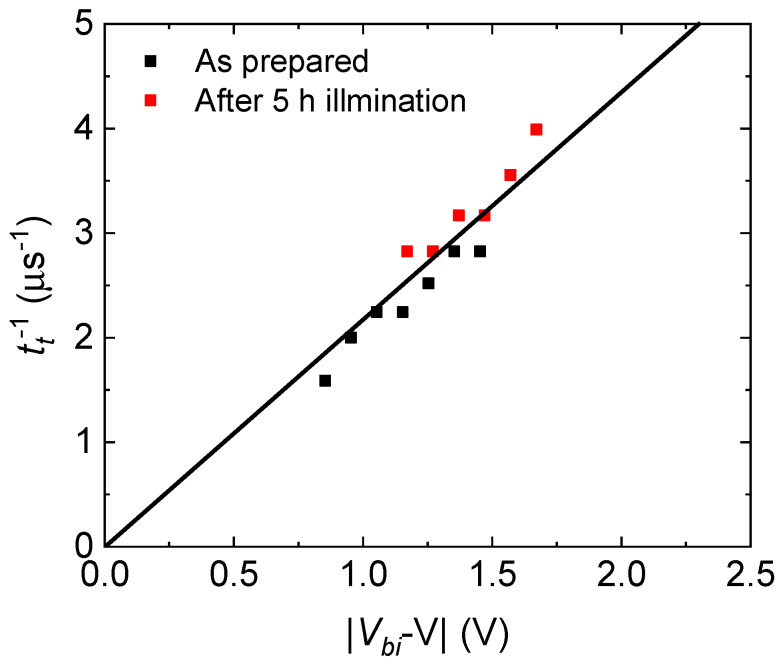
Effective applied voltage dependency of inverse transit times calculated from Figure 11 before and after 5 h of photoinduced degradation.

To gain insight into the degradation mechanisms of PTB7:PC_71_BM OPVs with PTB7 content of 40 wt%, we carried out MPC measurements before and after AM1.5G light soaking. Figure 11 shows MPC spectra under different biasing conditions before and after light soaking for 5 h. Single peaks around 1 MHz and shoulders around 10 kHz are seen. The frequencies of the shoulders do not depend on applied voltage, reflecting that the shoulders are not due to the transit of charge carriers. The inverse transit times calculated from the frequencies at the peaks are proportional to the effective applied voltages, as shown in Figure 12, and the inverse transit times fall on the same straight line before and after AM1.5G light soaking. The results show that the values of electron and hole mobility are essentially not changed, and, in addition, electron and hole mobility are still well balanced after light soaking. Our device simulation shows that electron and hole mobility can be simultaneously (separately) determined by means of MPC spectroscopy when the electron mobility is 10 times higher or lower than the hole mobility, showing that the photoinduced change in mobility is very small in the present case. Figure 11 and Figure 12 demonstrate that MPC spectroscopy is applicable to the study of changes in the transport properties of working OPVs after light soaking.

### 4.5. Photoinduced Degradation Mechanism in OPVs

Although the electronic transport properties are not changed in PTB7:PC_71_BM OPVs after AM1.5G light soaking, the solar cell performance is degraded, as shown in Figure 9. We examined the incident photon to current conversion efficiency (IPCE) spectra before and after AM1.5G light soaking, and found that IPCE was reduced after light soaking, but the shapes of the IPCE spectra were not essentially changed, indicating that photoinduced decomposition or oxidation does not take place in PTB7:PC_71_BM BHJ [25,26]. It has been reported that a possible origin of the degradation in PTB7:PC_71_BM OPVs is the growth of PC_71_BM domains upon light soaking [27]. We believe that this is the case in the present study. The increase in PC_71_BM domain size reduced the interface area of PTB7 and PC_71_BM, leading to reduced photocarrier generation efficiency (hence, J_sc_). Photocarrier generation efficiency was measured from the reverse bias J–V characteristics under AM1.5G irradiation [28], and were reduced from 9.1 × 10^21^ cm^−3^s^−1^ to 6.6 × 10^21^ cm^−3^s^−1^. We point out here that the slight decrease in V_oc_ caused by AM1.5G light soaking in Figure 10 is due mainly to the decreased photocarrier generation efficiency, because the bimolecular recombination constant γ was not strongly affected by light soaking and was 3.1 × 10^−11^ cm^3^s^−1^ and 1.2 × 10^−11^ cm^3^s^−1^ before and after light soaking, respectively (the bimolecular recombination constants were measured by an open circuit photovoltage decay experiment [29]).

The increase in PC_71_BM domain size did not affect electron and hole mobility. Hole mobility was originally insensitive to PC_71_BM content, as shown in Figure 8, and it is likely that it was insensitive to the domain size as well. Electron mobility was also insensitive to the domain size of PC_71_BM. We carried out a Monte Carlo simulation for percolative hopping transport in a simple cubic lattice (hopping sites were placed on lattice points with certain occupation probability) to explain the transient transport properties [23], studied by means of time-of-flight transient photocurrent experiments in molecularly doped polymers, which are formed by the doping of holes or electrons transporting small molecules to electrically inactive polymer binders such as polystyrene and polymethylmethacrylate. The molecularly doped polymers are an important class of materials for the model percolative hopping system and for photoreceptors in electrophotographic applications. We found that simulated drift mobility abruptly increased just above the percolation threshold and slightly increased well above the threshold. The growth of PC_71_BM domains induced by AM1.5G light soaking can be regarded as the increased size of clusters of dense hopping sites on the lattice points well above the percolation threshold, and the simulated drift mobility was not greatly dependent on the configuration of the clusters at constant occupation probability, which is well above the threshold. The electron transport of PTB7:PC_71_BM BHJ with PTB7 content of 40 wt%, which is well above the percolation threshold [19], is therefore insensitive to the domain size of PC_71_BM.

In addition to the decrease in J_sc_, the reduction in FF contributes almost equally to the degradation of photovoltaic performance of PTB7:PC_71_BM OPVs. First, we examine the influence of transport properties on FF before and after AM1.5G irradiation. The transport properties are closely related to FF; the competition between charge extraction and recombination is expressed as a single parameter *θ*, and it was demonstrated from the experiments and device simulation of OPVs that this parameter is directly related to FF [30], and is defined as

(5) $$\theta =\frac{\gamma GL^{4}}{\mu_{n}\mu_{p}V_{bi}^{2}}$$

Parameter *θ* was slightly reduced after AM1.5G irradiation (we used the values of V_oc_ instead of *V_bi_*) mainly because of the reduction in *G*. According to [30], a slight reduction in parameter *θ* leads to improvement of FF, indicating that changes in the transport properties of PTB7:PC_71_BM OPVs induced by AM1.5G irradiation are not the origin of the reduction in FF.

Then we carried out the equivalent circuit analysis based on a one-diode model [31] to examine the relationship between FF and the equivalent circuit components. We fitted the following expression to J–V characteristics in Figure 9:

(6) $$J=J_{s}\left[\exp\left\{\frac{q(V-JR_{s})}{nkT}\right\}-1\right]+\frac{V-JR_{s}}{R_{sh}}-J_{light}$$

where *J_s_* is the reverse saturation current, *q* is the electronic elementary charge, *n* is the diode ideality factor, *k* is the Boltzmann constant, *R_s_* is the series resistance, *R_sh_* is the shunt resistance, *T* is the temperature, and *J_light_* is the photocurrent. The fitting was done using a combination of the genetic algorithm (GA) and Levenberg–Marquardt algorithm (LMA) [31].

LMA has been widely used to solve nonlinear least squares problems (curve-fitting problems) [32]. LMA is a gradient-based search algorithm and is very powerful once the starting point is close to the global minimum. Thus, choosing the initial point is very important for the algorithm. If the initial point is not appropriate, then LMA finds only a local minimum. On the other hand, GA mimics the natural selection and evolution process and is a nongradient algorithm [33,34]. After relatively lengthy computation time, GA generates a suboptimal result from the whole search space (in the present case, the whole search space of the components of the equivalent circuit in Equation (6)). Compared to LMA, given enough evolution time, GA is more likely to produce a result near the global minimum and find the global minimum after extremely lengthy computation time. If the result near the global minimum found by GA is used as the starting point of LMA, the global minimum can be found easily. The combination of LMA and GA can thus find a global minimum after acceptable computation time.

The drastic changes caused by AM1.5G irradiation over 5 h are reductions in both *J_light_* (11.9 to 8 mAcm^−2^) and *R_sh_* (96.0 to 10.6 kΩcm^2^), while the other parameters are almost unchanged (*J_s_* = (9.0 − 12) × 10^−5^ mAcm^−2^, *R_s_* = 20.7 − 24.2 Ωcm^2^, and *n* = 2.29 − 2.52). It has been known that a reduction in *R_sh_* causes a reduction in FF [35,36], and in the present case, the reduction in FF of PTB7:PC_71_BM inverted OPVs caused by AM1.5G irradiation is due to the reduction in *R_sh_*. It is likely that the reduction in *R_sh_* is caused by the formation of a shunting path in ZnO during AM1.5G irradiation [37,38]. The formation of the shunting path is a unique degradation process in inverted OPVs, in which ZnO thin films are coated onto ITO substrates as electron transport layers.

We briefly discuss possible ways of mitigating the degradation in inverted PTB7:PC_71_BM OPVs. As mentioned above, the degradation is likely due to the increased size of PC_71_BM domains in PTB7:PC_71_BM BHJ and the appearance of shunting paths in ZnO. Chemical modification of fullerene [39] in BHJ may be a way to suppress the changes in morphology of BHJ, i.e., the size of PC_71_BM before and after AM1.5G irradiation. Newly developed amorphous oxide alloys such as InGaO and GaZnSnO [40] may not form shunting paths by AM1.5G irradiation. A study investigating the photoinduced degradation of inverted OPVs with such an electron transport layer instead of ZnO would be valuable. In addition, the formation of a polyethyleneimine layer with a thickness of several nanometers onto ZnO is another way to suppress the shunting path, because it is expected that the polyethyleneimine layer passivates the shunting paths in ZnO [41,42].

## 5. Conclusions

We studied the electronic transport properties of working PTB7:PC_71_BM OPVs with inverted configuration using MPC spectroscopy before and after AM1.5G irradiation. The photovoltaic performance (J_sc_, V_oc_, FF, and PCE) of PTB7:PC_71_BM OPVs with different PTB7 content was characterized from their J–V characteristics, and the best power conversion efficiency was obtained at PTB7 content of 40 wt%, consistent with the literature. Electron and hole mobility was determined with MPC spectroscopy in working PTB7:PC_71_BM OPVs, and the overall behavior of their PTB7 content dependency was consistent with that measured in EODs and HODs of PTB7:PC_71_BM BHJ with the SCLC technique. AM1.5G irradiation to PTB7:PC_71_BM OPVs at PTB7 content of 40 wt% for 5 h degraded PCE, due mainly to the reduction in J_sc_ and FF to 70% and 72% of their initial values, respectively, and caused almost the same degradation of the photovoltaic performance of PTB7:PC_71_BM OPVs under open and short circuit conditions. MPC spectroscopy was carried out on degraded PTB7:PC_71_BM OPVs. AM1.5G irradiation did not cause the changes in mobility and hence the degradation was not due to changes in the electronic transport properties. The degradation of the OPVs was due mainly to the growth in PC_71_BM domains and the formation of shunting paths in ZnO.

## References

[1] Yang Y., Li G. (2015). Progress in High-Efficient Solution Process Organic Photovoltaic Devices Fundamentals, Materials, Devices, Devices and Fabrication.

[2] Zhang H., Yao H., Hou J., Zhu J., Zhang J., Li W., Yu R., Gao B., Zhang S., Hou J. (2018). Over 14% Efficiency in Organic Solar Cells Enabled by Chlorinated Nonfullerene Small-Molecule Acceptors. Adv. Mater..

[3] Hou J., Inganas O., Friend R.H., Gao F. (2018). Organic solar cells based on non-fullerene acceptors. Nature Mater..

[4] Kotlarski J.D., Blom P.W.M. (2012). Impact of unbalanced charge transport on the efficiency of normal and inverted solar cells. Appl. Phys. Lett..

[5] Shrotriya V., Yao Y., Li G., Yang Y. (2006). Effect of self-organization in polymer/fullerene bulk heterojunctions on solar cell performance. Appl. Phys. Lett..

[6] Morii K., Ishida M., Takashima T., Shimoda T., Wang Q., Nazeeruddin M.K., Grätzel M. (2006). Encapsulation-free hybrid organic-inorganic light-emitting diodes. Appl. Phys. Lett..

[7] Kyaw A.K.K., Sun X.W., Jiang C.Y., Lo G.Q., Zhao D.W., Kwong D.L. (2008). An inverted organic solar cell employing a sol-gel derived ZnO electron selective layer and thermal evaporated MoO_3_ hole selective layer. Appl. Phys. Lett..

[8] Hau S.K., Yip H.-L., Jen A.K.-Y. (2010). A Review on the Development of the Inverted Polymer Solar Cell Architecture. Polym. Rev..

[9] Reale A., La Notte L., Salamandra L., Polino G., Susanna G., Brown T.M., Brunetti F., Di Carlo A. (2015). Spray Coating for Polymer Solar Cells: An Up-to-Date Overview. Energy Technol..

[10] Kopola P., Aernouts T., Sliz R., Guillerez S., Ylikunnari M., Cheyns D., Välimäki M., Tuomikoski M., Hast J., Jabbour G. (2011). Solar Energy Materials & Solar Cells. Sol. Energy Mater. Sol. Cells.

[11] Hübler A., Trnovec B., Zillger T., Ali M., Wetzold N., Mingebach M., Wagenpfahl A., Deibel C., Dyakonov V. (2011). Printed Paper Photovoltaic Cells. Adv. Energy Mater..

[12] Välimäki M., Apilo P., Po R., Jansson E., Bernardi A., Ylikunnari M., Vilkman M., Corso G., Puustinen J., Tuominen J. (2015). R2R-printed inverted OPV modules—Towards arbitrary patterned designs. Nanoscale.

[13] Fukuda T., Takagi K., Asano T., Honda Z., Kamata N., Ueno K., Shirai H., Ju J., Yamagata Y., Tajima Y. (2011). Bulk heterojunction organic photovoltaic cell fabricated by the electrospray deposition method using mixed organic solvent. Phys. Status Solidi RRL.

[14] Jørgensen M., Norrman K., Krebs F.C. (2008). Stability/degradation of polymer solar cells. Sol. Energy Mater. Sol. Cells.

[15] Reese M.O., Nardes A.M., Rupert B.L., Larsen R.E., Olson D.C., Lloyd M.T., Shaheen S.E., Ginley D.S., Rumbles G., Kopidakis N. (2010). Photoinduced degradation of polymer and polymer-fullerene active layers: Experiment and theory. Adv. Funct. Mater..

[16] Kawano K., Pacios R., Poplavskyy D., Nelson J., Bradley D.D.C., Durrant J.R. (2006). Degradation of organic solar cells due to air exposure. Sol. Energy Mater. Sol. Cells.

[17] Naito H., Iwai T., Okuda M. (1991). A simple microcomputer-based modulated photocurrent spectroscopy system for the measurement of localized-state distributions in amorphous semiconductors. Meas. Sci. Technol..

[18] Nojima H., Kobayashi T., Nagase T., Naito H. (2019). Modulated Photocurrent Spectroscopy for Determination of Electron and Hole Mobilities in Working Organic Solar Cells. Sci. Rep..

[19] Ho C.H.Y., Cheung S.H., Li H.-W., Chiu K.L., Cheng Y., Yin H., Chan M.H., So F., Tsang S.-W., So S.K. (2017). Using Ultralow Dosages of Electron Acceptor to Reveal the Early Stage Donor–Acceptor Electronic Interactions in Bulk Heterojunction Blends. Adv. Energy Mater..

[20] Martens H.C.F., Huiberts J.N., Blom P.W.M. (2000). Simultaneous measurement of electron and hole mobilities in polymer light-emitting diodes. Appl. Phys. Lett..

[21] Ishihara S., Hase H., Okachi T., Naito H. (2011). Bipolar carrier transport in tris(8-hydroxyquinolinato) aluminum observed by impedance spectroscopy measurements. J. Appl. Phys..

[22] Takada M., Nagase T., Kobayashi T., Naito H. (2019). Full characterization of electronic transport properties in working polymer light-emitting diodes via impedance spectroscopy. J. Appl. Phys..

[23] Ogawa N., Naito H. (2002). Transient hopping transport in percolation clusters. Electr. Eng. Japan.

[24] Upama M.B., Wright M., Veettil B.P., Elumalai N.K., Mahmud M.A., Wang D., Chan K.H., Xu C., Haque F., Uddin A. (2016). Analysis of burn-in photo degradation in low bandgap polymer PTB7 using photothermal deflection spectroscopy. RCS Adv..

[25] Mamada M., Kumaki D., Nishida J., Tokito S., Yamashita Y. (2010). Novel Semiconducting Quinone for Air-Stable n-Type Organic Field-Effect Transistors. ACS Appl. Mater. Interfaces.

[26] Kettle J., Ding Z., Horie M., Smith G.C. (2016). XPS analysis of the chemical degradation of PTB7 polymers for organic photovoltaics. Org. Electron..

[27] Jeong J., Seo J., Nam S., Han H., Kim H., Anthopoulos T.D., Bradley D.D.C., Kim Y. (2016). Significant Stability Enhancement in High-Efficiency Polymer: Fullerene Bulk Heterojunction Solar Cells by Blocking Ultraviolet Photons from Solar Light. Adv. Sci..

[28] Blom P.W.M., Mihailetchi V.D., Koster L.J.A., Markov D.E. (2007). Device Physics of Polymer:Fullerene Bulk Heterojunction Solar Cells. Adv. Mater..

[29] Pisarkiewicz T. (2004). Photodecay method in investigation of materials and photovoltaic structures. Opto-Electron Rev..

[30] Bartesaghi D., Pere I.C., Kniepert J., Roland S., Turbiez M., Neher D., Koster L.J.A. (2015). Competition between recombination and extraction of free charges determines the fill factor of organic solar cells. Nature Commun..

[31] Nishida K., Oka M., Hase H., Naito H. (2011). Determination of Physical Parameters in Organic Bulk Heterojunction Solar Cells Using a Genetic Algorithm. Trans. IEEJ C.

[32] Marquardt D.W. (1963). An algorithm for least-squares estimation of nonlinear parameters. J. Soc. Ind. Appl. Mathemaitcs.

[33] Goldberg D. (1989). Genetic Algorithms in Search, Optimization, and Machine Learning.

[34] Deb K. (2001). Multi-Objective Optimization Using Evolutionary Algorithms.

[35] Jao M.-H., Liao H.-C., Su W.-F. (2016). Achieving a high fill factor for organic solar cells. J. Mater. Chem. A.

[36] Qi B., Wang J. (2013). Fill factor in organic solar cells. Phys. Chem. Chem. Phys..

[37] Kam Z., Wang X., Zhang J., Wu J. (2015). Elimination of Burn-in Open-Circuit Voltage Degradation by ZnO Surface Modification in Organic Solar Cells. ACS Appl. Mater. Interface.

[38] Manor A., Katz E.A., Tromholt T., Krebs F.C. (2011). Electrical and Photo-Induced Degradation of ZnO Layers in Organic Photovoltaics. Adv. Energy Mater..

[39] Fernandez D., Viterisi A., Ryan J.W., Guirado F.G., Vidal S., Filippone S., Martin N., Palomares E. (2014). Small molecule BHJ solar cells based on DPP(TBFu)_2_ and diphenylmethanofullerenes (DPM): Linking morphology, transport, recombination and crystallinity. Nanoscale.

[40] Zhou N., Kim M.-G., Loser S., Smith J., Yoshida H., Guo X., Song C., Jin H., Chen Z., Yoon S.M. (2015). Amorphous oxide alloys as interfacial layers with broadly tunable electronic structures for organic photovoltaic cells. PNAS.

[41] Liang Z., Zhang Q., Jiang L., Cao G. (2015). ZnO cathode buffer layers for inverted polymer solar cells. Energy Environ. Sci..

[42] Takada M., Nagase T., Kobayashi T., Naito H. (2017). Electron injection in inverted organic light-emitting diodes with poly(ethyleneimine) electron injection layers. Org. Electron..

